# Perceptions of the Self Versus One’s Own Social Group: (Mis)conceptions of Older Women’s Interest in and Competence With Technology

**DOI:** 10.3389/fpsyg.2020.00848

**Published:** 2020-05-19

**Authors:** Alina Gales, Sylvia V. Hubner

**Affiliations:** ^1^TUM School of Governance, Technical University of Munich, Munich, Germany; ^2^Department of Management and Organisation, NUS Business School, National University of Singapore, Singapore, Singapore

**Keywords:** gender roles, intersectionality, power/social status, prejudice/stereotyping, social media, self-perception

## Abstract

Our analysis investigates how gender, age, and technology stereotypes relate to one another and how this relationship reinforces or questions stereotypes. Based on intersectionality, stereotyping, and sense-making literature, our study explores how older women perceive their own interest in and competence with technology and that of their peers. We conducted qualitative in-depth interviews with women between 65 and 75 years of age in Germany. Our findings indicate that their evaluations of others are age and gender stereotyped. When explaining their own interest in technology, they refer to their individual preferences, and for explaining their own competence of technology, they refer to social categories. Plus, assumptions of technology usage seem to be gendered. On the basis of our findings, we discuss the need for taking social categories into account when evaluating inclusiveness with new technologies.

## Introduction: the Connections of the Social Categories of Gender and Age With Technology

A survey in Great Britain found that “individuals are overwhelmed by the power and potential of the changes new technologies bring” (Doteveryone, 2018). When examining the connections and associations of gender and technology, the common assumption is to evaluate it positively for men and negatively for women (Balsamo, 2014; Girls Who Code, 2019). With regard to age and technology stereotypes, older individuals are usually perceived as less competent than younger people (Loe, 2010). With this in mind, how does a group of people seemingly most affected by these clichés – older women^1^ – feel about such stereotypes? What is more: How do older women assess themselves when it comes to gender and technology as well as age and technology? For a nuanced understanding of the reasons and mechanisms underlying the perceptions of new technologies in different individuals, more knowledge about perceptions of technology usage, particularly in relation to age and gender stereotypes, is needed.

Based on intersectionality, stereotyping, and sense-making literature, our study investigates how older women perceive their own interest in and competence with technology as well as that of their peers. Women seem to self-stereotype more firmly than men (Cadinu and Galdi, 2012). Gender and technology stereotypes demonstrate a negative connection for women (Balsamo, 2014; Starr, 2018). Age and technology stereotypes seem to view older people as being less competent with technology (Zeljko, n.d.). These stereotypes can lead to biased perceptions about one’s own interest and competence as well as that of others. The concept of intersectionality considers situations in which certain combinations of social categories play together. Certain combinations exacerbate power relations and lead to particular discrimination (Crenshaw, 2017). Hence, it is important to understand how members of a group falling victim to several categories of stereotyping make sense of their own experiences and behaviors in relation to these common perceptions (Pirolli and Card, 2005).

To what extent does it matter how and why people use technologies? Technology is used and perceived in different ways by different people. There are various forms of “action identification” (Bouwhuis, 2000, p. 908), i.e., what do users themselves think that they are doing? Individuals might use technology for entertainment, with a goal in mind, rationally, or with feelings involved. Either way, some forms of usage appear to be intuitive, whereas other ways can become challenging (Bouwhuis, 2000), and for some people more than for others.

Drawing on qualitative in-depth interviews with women between 65 and 75 years old, we analyzed these women’s interest in and perceived competence with technology as well as their subjective reasoning, particularly with respect to age and gender. We interviewed women between 65 and 75 years old because both their gender and their age are negatively judged with regard to technological interest and competence (McLaughlin et al., 2012; Girls Who Code, 2019). Based on these interviews, our study discovered when they repeat common beliefs regarding gender, age and technology, and when they depart from the stereotypes.

Our study’s contributions to the literature are twofold: first, we lay bare how older women seem to view their unique personal setup as reason for why they are interested in and use technology, while their perceptions of others are likely to be based on either stereotypes and social norms (gender relates to interest) or inferences from their own experiences (age relates to competence). Second, we reveal when older women refer to prevalent clichés in society of what they call female and male technology related interest. While individual preferences usually are a preferred explanation, some stereotypes can come in handy to justify own behaviors (age stereotypes for competence). Our findings can inform politicians and also designers behind the technology because they influence how products are used and, therefore, who finds it challenging, and who finds it interesting, and who does not (Maass and Rommes, 2007).

## Theory: Technology Is Tied to Social Structures

Technologies are ultimately interwoven with people, so they can be read in an anthropological and historical way – with respect to people having always created instruments of utility as well as the evolving progresses made with these tools (Lerman et al., 2003). Understandably, words to describe human interaction with technology are all verbs of activity: “making, doing, using, designing, producing, consuming, repairing, recycling” (Lerman et al., 2003, p. 3). Technological products are not just artifacts that naturally happen to be in our world with people making use of them for whichever reasons. As technological products are created by people from scratch, they are automatically interwoven in a societal, historical, and economical context: “Technologies embody and advance political interests and agendas, the product of social structure, culture, values, and politics as much as they are the result of objective scientific discovery” (Wajcman, 2006, pp. 17–18).

Due to the increasing societal role of various forms of technology, and specifically digital technology, we consider, for this study, technology to include not only technological devices (such as smartphones, laptops, notebooks, computers, wearables, e-readers, etc.) but also services (apps, streaming platforms, shared services, etc.), digital communication forms (social networks and social media), and data (definition based on the D21 Digital Index 2019/2020, an overview of the digital situation in Germany) (Initiative D21, 2020).

### Technology as an Additional Category of Intersectionality

We drew on intersectionality as a framework for understanding the mutual influence of the social categories of gender and age with technology. The term “intersectionality” was originally coined by Crenshaw in 1989; her updated definition states that “intersectionality is a lens through which you can see where power comes and collides, where it interlocks and intersects” (Crenshaw, 2017). Each social inequality has a different and unique form of inclusion and exclusion that is created in relation to a certain idea of “normal,” i.e., that of a socially accepted or powerful group of people. Power refers to the individual availability of the resources relevant to society, which are not equally distributed. Power relations depend on gender and age. For older women, gender and age disadvantages are combined, making them a rather powerless group. Individuals do not necessarily need to identify with a social category for it to become a part of intersectional analysis, nor does this category need to be naturally given to be perceived as a power component.

We argue that technology adds another layer that influences power relations in social situations. Therefore, we suggest that intersectionality opens up the possibility of viewing technology as a new component of intersectionality. Technology is part of our daily life – to a varying extent – and it creates hierarchies, felt or not (The Digital Divide, 2019). We follow the perspective of Castells et al. (2007, p. 75) who suggest that technology is “practiced” and therefore is society and embodies society.

Technology has been declared a social component and is, in combination with gender, an increasingly common research interest (Costa and Feltrin, 2016). The rise of digital technologies in recent years has created unlimited possibilities, developments, and scenarios and has resulted in a debate about the distribution of technology and decision-making regarding technology. When technologies are recognized as a power component, they are an aspect of social situations that potentially create intersectionality. A few researchers have listed technology even as aspects of intersectionality (Lykke and Hearn, 2010). Referring to De Vita et al. (2016, p. 510), who see “intersectionality as a dynamic site constructed in practice as well as in the tension between risks of discrimination and construction of opportunities,” we here investigated technology as a social component that can add on the intersectionality created by age and gender.

### Stereotypes as a Foundation for Perceptions of Technology Usage

To understand perceptions of the connection between gender and technology as well as age and technology, we need to consider the respective stereotypes, because stereotypes can contain (mis)conceptions of older women’s interest in and competence with technology. Stereotypes serve as a common reference in daily conversations. It is “a summary characterization of a human group, usually arising from and fortifying prejudices for or against that group, and used as a template into which individual members of the group are made to fit. Stereotyping is probably a necessary element in any attempt to cope with groups of which one is not a member, but the possibilities of injustice to which it gives rise are now all too familiar” (Scruton, 2007, p. 665).

Stereotypes can be prescriptive, i.e., depicting what someone should do, and descriptive, i.e., depicting what someone typically does (Koenig, 2018). Given our analysis of perceptions regarding general technology usage, we focused on the descriptive nature of stereotypes that are illustrative of convictions regarding how behavior typically is (Koenig, 2018). Clichés become evident in stereotypes and reinforce power relations, as explained by intersectional analysis. Importantly, stereotypes associated with a certain group of people might differ tremendously from the way in which individuals within that group identify themselves personally (Hentschel et al., 2019).

#### Stereotypes of Gender and Technology: Positively for Men and Negatively for Women

“Men are traditionally identified as the idealized and most important agents of technological development, while women are cast as either unfit, uninterested, or incapable” (Balsamo, 2014, p. 20). This quote sums up the stereotyped and gendered perception of technology in society. Maass and Rommes (2007) argue that gender and technology cannot be separated from each other because technology reinforces the existing gender relations in society. Such stereotypes also become evident when new technologies represent certain characteristics typically associated with the female or male gender. Specifically, gender stereotypes of men as innately talented with anything related to math, science, and physics (Nurlu, 2017) and women as typically playing a communicative and caring role (Bauer, 2015; Wright, 2017) are of significance to this study. Furthermore, even “if designers have been unaware of gender, or gender-blind, they may unconsciously design for the male norm in society, leaving out or making invisible feminine connotated elements of the work or of work done by women in general” (Maass and Rommes, 2007, p. 98). Hence, gender and technology stereotypes seem to not only appear in people’s minds. They are also inscribed in technological objects (Tallon, 2019). Therefore, it can be understood that the development of technology and the social shaping of gender with the aforementioned stereotypes have progressed in parallel (Paulitz and Prietl, 2014).

Looking at the numbers, statistics from the D21 Digital Index (2016, p. 19) show that, in comparison to men, women have much less knowledge in relation to digital literacy – up to 21 percentage points of difference. An exploration of women’s participation in the educational and professional contexts of technology shows that they are missing out on equal access and opportunities (BarNir, 2012). Women studying engineering not only have to face stereotypes of being less proficient than men, but they also feel uncomfortable when conversing about that cliché (Kronberger and Horwath, 2013). An extensive study by Ihsen et al. (2014) finds that, even though women are socially integrated into degree programs in the areas of math, computer science, natural sciences, and technology, when they have a job (if not sooner), they have to further prove themselves more than men in order to be accepted by their peers and respected for their professional knowledge. Plus, in the professional technology sector, women are paid less than men and work in lower positions (Ranga and Etzkowitz, 2010). Therefore, perceptions of technology usage and competence differ according to gender (Kuchynka et al., 2018).

#### Stereotypes of Age and Technology: Positively for Younger People and Negatively for Older People

When it comes to age and technology, older people are not typically associated with technological competence in comparison to younger people, who are perceived as tech-savvy (Zeljko, n.d.). Older people are usually not included in the design processes since digital technologies are not created with older people in mind (Loe, 2010). What is more, older people apparently repeat stereotypes about their perceived level of digital literacy, as a study by McLaughlin et al. (2012) shows. In this study, older participants noted that they feel like they should not enjoy playing video games, nor should they have the ability to do so. In another recall test, Stine-Morrow et al. (2006) find that, compared to younger people, older people do not make use of their cognitive capacities as much as they are able to due to lesser “memory self-efficacy that limit[s] the recruitment of resources that are available (‘what you believe you can do’)” (p. 801). Such findings could relate to the circumstance that older age groups did not grow up with digital technologies and might have difficulties getting access to and feeling comfortable with using them (De Schutter and Vandenabeele, 2008).

Numbers underpin the observation that older people are less experienced with computers than their younger counterparts (Bolle et al., 2015). Even though the internet has become increasingly popular with older people (Initiative D21, 2016), they evaluate it as being less relevant to them than it is for younger age groups: only 11–13% of people aged 60 years or older in Germany state that it would have negative consequences on their lives if the internet disappeared (Initiative D21, 2016, p. 20). However, people in their 60s in Germany represent the age group having the strongest belief that it is necessary to provide digital media in schools and to have students learn how to code (Initiative D21, 2016, p. 20). This number might hint at older people’s awareness of the current need for knowledge of and competence with digital technology. Taken together, how people are perceived with regard to technological usage and competence also depends on people’s estimated age (Ball et al., 2017). Plus, people’s access to technology is tied to their age.

Stereotypes of gender and age and technology “reinforce existing understandings of old women as unimportant, old-fashioned, homebound, lonely and child-like” (Mosberg Iversen, 2015, para. 8), and stereotypes frame women and older people as uncharacteristic users, experts, or owners of technology.

### Sense-Making and Biases as a Basis for Evaluating the Self and Others

Given that people often have a hard time organizing and evaluating the complexity of information, they form perceptions and evaluations based on biases (Pirolli and Card, 2005). In sense-making processes, people typically refer to “existing schemas and existing expectations” (Pirolli and Card, 2005, para. 3.2) in order to form evaluations in line with common beliefs and to ensure confirmation because finding and making refuting statements would take much more effort. People often base their decisions on quick estimations of existing and common beliefs.

Interestingly, Pronin et al. (2004) found that people believe they fall prey to biases less easily than others. People tend to think that they are personally free of biases in their judgments. Even more so, they are inclined to believe that they are objective in comparison to others, to whom they attribute subjectivity (Pronin et al., 2002).

When evaluating attitudes of others, the assessment is usually based on the other person’s behavior, even when there is also contextual information about what could possibly influence that person’s actions. Actually, people have a tendency to overly emphasize a person’s behavior in evaluating that individual’s attitude (Jones and Harris, 1967), underestimating external factors; this is referred to as correspondence bias (Gilbert and Malone, 1995). The explanation for a judgment of other people’s behavior then is connected to observations made over time. Thus, evaluations of others’ interest in and usage of technology are likely to be based on observed behaviors, without considering the context of those observations.

Individuals’ declarations about their own attitudes are based on a construct they build of their selves assessed through “their own attitudes, emotions, and other internal states partially by inferring them from observations of their own overt behavior and/or the circumstances in which this behavior occurs” (Bem, 1972, p. 5). The self-perception theory of Bem (1972) proposes that people judge their internal being on their behavior. Hence, the self-perception of women’s interest in and usage of technology is influenced by their observations of their behaviors, including interactions with their surroundings.

As behavior usually is equally observable by oneself and others, both can guess the innate state, because both parties are watching the same action. Importantly, however, observations and perceptions of both the self and others are subjective and potentially biased.

In sum, we are relying on an intersectional framework for our focus on the connection between gender, age, and technology. Adding the concepts of stereotyping and sense-making builds the theoretical foundation of this study’s research interest. Our study aims at a comprehension of older women’s perspective on technology usage, of themselves, and of their peers. This way, we answer the call by McCormick-Huhn et al. (2019) to include an intersectional viewpoint in participants’ profiles to understand them on a more thorough level. More knowledge on older women’s perspective is necessary in order to understand the social structures forming discriminative positions. In our context, we look at technology’s accessibility for older women due to their gender and due to their age.

## Materials and Methods

### Participants

We conducted individual, qualitative in-depth interviews and observations on technology with 20 retired women born between 1943 and 1953 and living in the southwest of Germany. In Table 1, we present a detailed overview of the participants’ year of birth, age at the time of the interview, year of retirement, former occupation, current use of technologies, the technologies they used at their former job, their educational background, and relationship status. All of the women were between 65 and 75 years old, and none of them was employed. All interviewees were selected from the same region in order to warrant a comparable language practice and a similar socioeconomic surrounding. Apart from that, their year of retirement ranges from 1991 to 2017, and most of them are married, though some are widowed and a few are divorced or had never married. Their former job areas include administrative work, teaching positions, and manually operating professions. Therefore, some occupations required the knowledge of distinguished computer programs while others used the computer as a typewriter. Some of the interviewed women did not have contact with technologies in their former job. At the point of the interview, most interviewees possessed a smartphone, only a few had a mobile phone, and some also owned a computer or a laptop or a notebook.

**TABLE 1:** Participant overview and details.

**Birth year**	**Age**	**Retirement**	**Relationship status**	**Educational background**	**Former occupation/profession**	**Technology at former job**	**Technology used today at home**
1948	70	2011	Widowed	Secondary school certificate	Seamstress and later a worldwide trainer for other seamstresses	Technical machines + mobile phone usage	Smartphone, laptop
1951	67	2001	Married	Minimal/compulsory school certificate	Secretary in a bank	Computer work	Smartphone, computer
1946	72	1997	Married	Minimal/compulsory school certificate	Hair stylist/hair dresser/hair colorist	None	Smartphone
1950	68	2013	Married	Minimal/compulsory school certificate	Learned in a bank, then worked as a market researcher	Different technical machines	Smartphone, computer
1950	68	2007	Married	Minimal/compulsory school certificate	Worked in husband’s farm	None	Smartphone
1950	68	2010	Married	Minimal/compulsory school certificate	Office secretary	Different computer programs	Smartphone, laptop, tablet
1950	68	2011	Married	Minimal/compulsory school certificate	Worked as a sales person in a clothing store	None	Mobile phone
1948	70	2008	Married	Minimal/compulsory school certificate	Assistant tax consultant	Computer work	Mobile phone
1952	66	2017	Single	Higher education: “Abitur” with a teacher education	Teacher	Laptop for school projects	Smartphone
1950	68	2015	Married	Secondary school certificate	First pharmaceutical technical assistant, then administrative support in husband’s company	Computer work	Smartphone, laptop
1947	71	1991	Married	Minimal/compulsory school certificate	Learned dressmaker, then worked in a kitchen	None	Mobile phone
1951	67	2015	Married but separated	Higher education: “Abitur” with a teacher education	Maths and physics teacher	Physical and mathematical understanding of technology	Smartphone, note pad and computer
1949	69	2011	Married	Minimal/compulsory school certificate	Worked on her father’s farm and then in a gardening and florist company	None	Smartphone
1948	70	2009	Married	Minimal/compulsory school certificate	Administrative tasks in family owned business	Computer as typewriter	Smartphone, e-reader
1945	73	2005	Divorced	Higher education: “Abitur” with a teacher education	Elementary school teacher	Computer as typewriter	Smartphone, note pad and computer
1943	75	2008	Widowed	Secondary school certificate	Trained bank administrative, then secretary	Computer as typewriter	Smartphone, laptop
1952	66	2016	Single	Secondary school certificate	Trained bank administrative, then secretary and self-employed	Computer programs	Smartphone, laptop
1952	66	2012	Married	Higher education: “Abitur” with a teacher education	Music teacher	Computer as typewriter	Smartphone and medical devices
1953	65	2015	Married	Minimal/compulsory school certificate	Worked in a lab in a producing company	For calculating only	Smartphone and laptop
1951	67	2016	Widowed	Minimal/compulsory school certificate	Worked as a board assistant	Computer work	Smartphone and laptop

### Interviews

The interviewer knew most of the interviewees personally through mutual points of contact or via referral from acquaintances. This circumstance promised to be beneficial to this study for a number of reasons: First, it helped to create a richer understanding of their social realities and what they find worth mentioning and leaving out. Second, it worked against the unfamiliarity of an academic study’s interview situation, to which the participants would be unaccustomed. Third, it was possible to visit the participants in their own homes to observe their technology usage in a private setting, which comes closer to their actual relation to the devices than in a public or unknown sphere. Visiting them also helped create a comfortable surrounding for the women, which was as normal as possible, in contrast to a staged research setting outside their homes. Fourth, the interviewees were able to speak in dialect and were therefore openly and unrestricted in their flow of speech.

The interviews were 20–95 min long and took place in the participant’s respective home. During the interview, interviewer and interviewee faced each other at a dining or kitchen table. The interviewer explained that the conversation will be recorded and transcribed, that anonymity is guaranteed, and that the exchange can be stopped by the interviewee at any time. All women agreed to be recorded on tape.

An interview guide gave directions in the interview but did not provide a strict set of questions. Interview questions referred to the participants’ own technology usage, competence, and evaluation and their perception about others’ technology usage, competence, and evaluation. Additionally, we asked questions about their life situation and what role technology plays in their daily routines. Based on the concept of semi-structured interviews (Whiting, 2008), the interviewer went loosely through the questions from beginning to end, but also asked additional questions based on the interviewees’ answers (Millwood and Heath, 2000). Moreover, the interviewer asked questions referring back to what the women had said (Whiting, 2008). Following qualitative in-depth interview principles, we aimed at creating an interview situation as close to a conventional conversation as possible. This procedure has been shown to enable an interview that goes beneath the surface of the prepared topics (Barriball and While, 1994).

Importantly, the interviewer left the definition of the term technology open for the interviewees. Our aim was to illuminate their own understanding of what technology is, without the interviewer restricting it beforehand. This way, we could analyze the participants’ personal opinion on technology. Please see the Appendix for the full interview guide.

The interviews were transcribed in German orthography without considering the spelling of the dialect but recording the exact spoken order of the words, even when contrary to German grammar. This means that the interviews were precisely transcribed in the German language verbatim, including all repetitions, hesitations, and disordered sentence structures and according to Höld’s (2009) guidance on the verbatim transcription of audio data. The statements that are representative for a category and that we wanted to include in the manuscript were translated into English. We also analyzed observational notes regarding the interviewees’ technology usage. As part of the interview, participants showed the interviewer a technological device of their choice (which was mostly their smartphone) and demonstrated how they use it. For example, some presented the apps they regularly use or the photos they recently took. This helped us in getting information on the apps and services the participants use and in deriving implications. For anonymity reasons, the identities of the interviewees were protected by using the letter “G” followed by an allocated number in all transcripts and in this manuscript.

After 20 interviews, we reached a point of redundancy because we found our interviewees to be repeating previously discovered concepts (Cleary et al., 2014). We reached a level of saturation where “the collection of new data does not shed any further light on the issue under investigation” (Mason, 2010, p. 2).

### Analysis

The analysis of the interviews was based on Braun and Clarke’s (2006) thematic analysis approach of “identifying, analyzing and reporting patterns (themes) within data” (Braun and Clarke, 2006, p. 79). Before deep diving into the transcriptions of the interviews, we decided to focus on the role of gender and age for perceptions of technology. Then, we started actively seeking themes with research relevance, based on the data. Thus, we opted for a theoretical thematic analysis in order to serve our research question (Braun and Clarke, 2006). After the transcription of the interviews, all texts were inductively coded with the MAXQDA 2018 program. First, we applied initial open coding by analyzing the transcriptions line by line, moving within the data. Then, we applied focused and selective coding, deciphering the most expressive, symbolic and condensed codes that eventually led us to the main categories, which we present in this manuscript. Following the idea of constructivist grounded theory, we made sure to not be stiff but remain open within the coding process (Thornberg and Charmaz, 2014). Following the thematic analysis idea, we aimed at laying bare the beliefs, understandings, and declarations of the interviewed women. As it is typically conducted within the latent level of analysis, we followed a constructivist perspective, in which we sought to organize the background of and reasoning behind those statements. By creating coding maps, we could continually check whether and how our data and codes worked with and within each other. In the next step, we applied an analysis that demonstrates the specific connections and relations for gender and technology as well as age and technology. Finally, we chose the data best representing the themes of relevance for answering the research question and analyzed them by referring back to the literature and theoretical framework presented above (Braun and Clarke, 2006).

## Results

### Evaluations of Interest in Technology Show a Discrepancy Between the Perspective of Oneself and Others

The women interviewed referred to various motives for their own and other people’s reasons for being interested in technology. It is striking that, when speaking of themselves, they based the root of their interest in technology on their individual preferences: “That is solely my, my being, my nature, my way” (G9). In contrast, when talking about others, they refer to gender as an explanation: “The boys were more interested. It doesn’t mean the girls didn’t get it, just the interest wasn’t there” (G12). In the following, we will elaborate on this discrepancy.

#### Own Interest in Technology Is Perceived to Stem From Individual Preferences

The older women referred to their individual preferences as the reasons for their interest in technology. Remarkably, this was true whether they thought they had a strong interest in technology or not. They did not state a further need to elaborate why or why not they had an interest in technology. Thus, the women described their own interest in technology as something that is entirely individual. When describing their own interest in technology, they tied technology to individuality rather than to social categories like age and gender: “As I said, technology is not *my* thing. I used to sit for many years, on the computer for many hours, did accounting, well financial accounting and payrolls and then I didn’t want to do it any longer at home [in retirement]” (G8).

When the interviewed women talked about the ways they use their technological devices, we could observe a broad variety of applications. For example, they buy medications online, solve crossword puzzles, do online banking, listen to music, read an e-book, use the smartphone or note pad for navigation or for video chats, among others. These examples suggest that the women indeed take ownership of digital technology, pleasing whatever interest they have in their life in general. In Table 2, we show quotes that give examples of the various ways how the women use technology.

**TABLE 2:** Interest in technology: discrepancy between perceptions of self versus others.

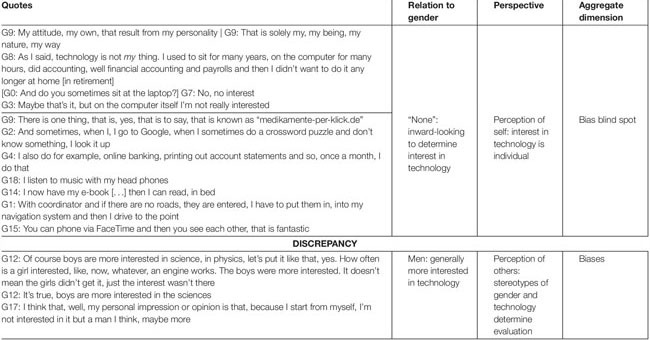

#### Men in General Are Seen to Be More Interested in Technology Compared to Women in General

Some of the women interviewed seemed to assign technical interest in general to men. Even more so, it starts as early as childhood, when certain interests in the sciences – and here, parallels to interest in technology were made by the women themselves – are seen as being more prevalent among boys: “Of course boys are more interested in science, in physics, let’s put it like that, yes. How often is a girl interested, like, now, whatever, an engine works” (G12). In Table 2, we demonstrate this discrepancy between the perception of oneself versus the perspective on others.

#### Men in General Are Associated With an Interest in Mechanical and Electrical Types of Technology

The women found a strong link between men and technology that can be paraphrased as mechatronic, electronic, mechanical, electrical, and such – therefore mainly in technical terms (see examples in Table 3). In this context, technology implies tools and machines that were initially designed to support or replace the physical power formerly needed by men. In this area, the women interviewed created the strongest connection to men, completely leaving out the possibility of women being interested in this kind of technology. Hence, interest in technology in relation to men was tied to the technical aspect thereof, which women are not perceived to be a part of: “I don’t know if a woman, for example, could repair a washing machine, right? That is, or a TV technician, I don’t know, are there women who are TV technicians?” (G6). Interestingly, there seems to be a distinction made here between speaking about the repair versus the use of technology; repair seems to be perceived to be male and rather questionable for women – which was not the case for the use of technology.

**TABLE 3:** Type of technology to be interested in, in relation to gender.

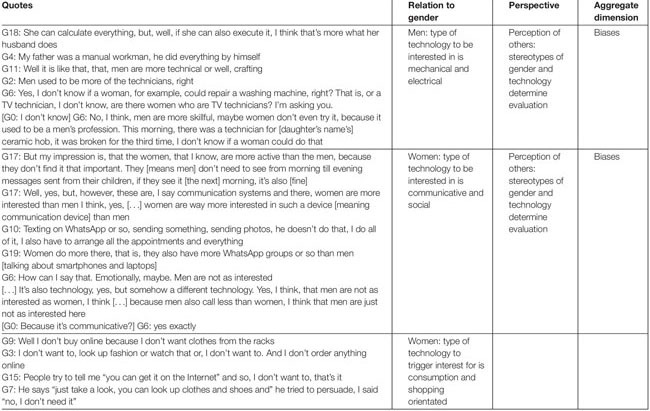

#### Women in General Are Associated With an Interest in Communicative and Shopping-Oriented Types of Technology

In contrast to the comments about men, the interest in technology related to women was linked to the social and communicative aspect, in which men were not included (see examples in Table 3). In this context, the caring and worrying characteristics of women were highlighted. Rooted in this aspect was a stronger interest in smartphones and laptops or computers. Again, technology seems to be an extension of gender stereotypes in society, the difference being that here, technology is not understood as a representation of the technically oriented man, but rather having a social aspect, e.g., writing e-mails and using apps for instant messaging like WhatsApp or social networks like Facebook. Checking in on friends and family members is considered to be a female task and something women enjoy doing more, which therefore leads to a stronger interest in making phone calls or texting others: “But my impression is, that the women, that I know, are more active than the men, because they don’t find it that important. They [men] don’t need to see from morning till evening messages sent from their children, if they see it [the next] morning, it’s also [fine]” (G17).

Another point worth noting are the multiple comments about buying clothes on the internet, which is presented twofold: First, some of the women mentioned online shopping as something they specifically do not do even though the interviewer did not ask them about it, which shows that online shopping has been a topic of conversation for the women before: “I don’t want to, look up fashion or watch that or, I don’t want to. And I don’t order anything online” (G3). Second, some women even particularly noted that the possibility of online shopping was presented to them by other people: “He says ‘just take a look, you can look up clothes and shoes and’ he tried to persuade, I said ‘no, I don’t need it”’ (G7). Here again, technology is representative of the female stereotype in society that women presumably love to go shopping.

When talking to the interviewed women about other people’s interest in technology, they resorted to gender references. They heavily relied on existing stereotypes of men and technology as well as of women and technology. In this context, it seems as if technology is an extension of the typical descriptions and categorizations of men and women found in society. Table 3 presents the types of technology people seem to be interested in, depending on their gender.

### Evaluations of Competence With Technology Show a Correspondence Between the Perspective of Oneself and Others

When it comes to the assessment of one’s competence with technology, the age factor becomes a reference point (see Table 4). The women interviewed indicated that age was a part of their identity and that age stereotypes provide reasoning for their perceived lack of technological competence. It seems to be an explanation that comes in handy for the women because using technology can create a challenge: “And then I always used to say ‘oh no, then I have to learn something all over again!”’ (G4). On this basis, an association is formed by the women, which is used to evaluate other people’s competence with technology because they are relying on such stereotypes of age and technology: “She is eight years older than me, she’s never had a computer […] she doesn’t know anything on the computer” (G10).

**TABLE 4:** Competence with technology: Correspondence of perceptions of self and others.

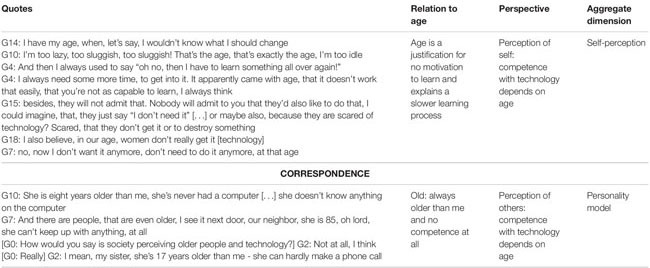

#### Age as a Justification for a Lack of Motivation to Learn and as an Explanation for a Slower Learning Process

For the interviewed women, it seems as if their seemingly advanced age is an explanation or even a justification for a lack of motivation to learn something new and generally explains a slower learning process (see examples in Table 4). For the women, being older means that there is a resistance to challenges; they try to avoid them and need more time to adjust to something new: “I always need some more time, to get into it. It apparently came with age, that it doesn’t work that easily, that you’re not as capable to learn, I always think” (G4).

#### The Perception of Others’ Competence With Technology Depends on Age

When the women spoke of other people’s competence with technology in relation to age, the word “old” meant inept. Technology stereotypes related to age are demonstrated equally well in this context. Interestingly, there was not a discrepancy between the women’s perception of themselves and their estimation of others, but rather a correspondence. The women seemed to build a bridge from their own experiences to the experiences they think even older people have. The interviewees found age to be a justification for other people’s lack of competence with technology: “My sister, she’s 17 years older than me – she can hardly make a phone call” (G2).

In the conversations with the older women about competence with technology, there were many references to stereotypes associated with age and technology, especially when providing reasons for their own behavior and that of others. Age and technology stereotypes were used to legitimatize the interviewees’ competence with technology and that of others. Table 4 exemplifies how age is an explanation for resistance to challenges and new learning.

## Discussion

In this study, we have demonstrated the ways in which older women base descriptions of themselves and others on their own specific being and experiences (Bem, 1972) and when they use social categories as a reference or an explanation (Kelley, 1973).

Our study contributes to the current literature in two different ways. First, when evaluating interest in technology, we show how older women distinguish between themselves and other people. On the one hand, we find that older women see their individual preferences as reasons for their technology usage, but, when talking about the interest in technology by women in general, and by other women, they refer to clichés. Thus, for themselves, they refer to the perception of their selves that influences their interest in technology. For others, however, their interest in technology is perceived to depend on gender. On the other hand, when relating age and technology, older women seem to infer from their own experiences when forming perceptions of others. They perceive competence with technology to decrease with age both for themselves and for others. In sum, older women seem to see their supposedly very own, very unique, and very individual self as reason for why they use technology, and their perceptions of other older women seem to be based on either stereotypes and social norms (gender relates to interest) or inferences from their own experiences (age relates to competence).

Second, reasons for technology usage appear to depict existing stereotypes of women and men in their society: with men, interest in and competence with technology seem to stem from natural competence and physical power. In this case, technology is mostly recognized as mechanical, electronic, mechatronic, and technical. We find quite the reverse for female-gendered technology stereotypes: for women, technology is ultimately seen in terms of social interaction (digital communication) and consumption (online shopping). With regard to age stereotypes, older women refer to their age when the subject is their lack of willingness to adapt their present use of analog technology to a digital world. When assessing willingness to change, older women seem to clearly distance themselves from younger people. Hence, age stereotypes appear to be a factor strongly influencing judgments of technological competence, not only for others but also for oneself, which is less true of gender stereotypes.

### The Gender and Technology Connection Shows a Discrepancy Between the Perception of Oneself and Others

The contrasting evaluations of interest in technology by the interviewed women exemplify how technology can be assessed in a variety of ways: the interviewees referred to individual preferences when talking about themselves and named various points of interest, such as buying medication online, searching for solutions to crossword puzzles, online banking, listening to music, using it for navigation or for video chats, as mentioned above. In contrast, they resorted to clichés when discussing the relationship between gender and technology in general as well as with respect to other people. It seems as if they distinguished between an inner and an outer view, i.e., a perspective derived individually or on a societal level. They seem to link the liking or disliking of technology to perceptions of their selves, but, when thinking of other women, there was a salient belief in a gender-related interest in technology based on assumptions reproduced time and again by society (Nurlu, 2017).

What is more, technology was framed as an extension of gender clichés, whereby usage by men is derived from natural competence and physical power. In this context, technology is mainly associated with objects mechanical, electronical, mechatronic, and technical, which we have linked to other studies on male stereotypes (Bieg et al., 2015; Nurlu, 2017). On the contrary, when thinking about technology and women, technology is ultimately connected to communication and consumption. In this case, the social aspect of digital technology is emphasized more as having fun while communicating is strongly associated with women and their use of technology. This is in line with stereotypes portraying women as communal and social beings (Hentschel et al., 2019). Again, technology is being viewed through the lens of an extension of gender clichés since other studies have shown that, typically, women are portrayed as people who enjoy communicating with each other but also taking care of others (Bauer, 2015; Wright, 2017). Another facet in which technological usage seems to be gendered is online shopping, which has been presented to the women by others as a potential motivation to go online and trigger an interest in technology. Based on our findings, we can reiterate the conclusions of other scholars who have also found that gendered stereotypes of men being assertive and women being communal are still prevalent (Hentschel et al., 2019). Our analyses show how these stereotypes are depicted in technology usage and its perception.

### The Bias Blind Spot as a Reason for Differing Evaluations of Interest in Technology in Oneself and in Others

Our findings on the bilateral viewpoints of older women regarding themselves and others accord well with studies on biases which state that

“… individuals have faith in the “realism” or objectivity of their own views, and are thus likely to assume bias on the part of those who fail to share those views. And it is this tendency to view others as influenced by bias that leads individuals to the conclusion that their opponents hold extreme and dogmatic points of view” (Pronin et al., 2002, p. 379).

When talking about their own interest in technology, older women take an inner look and emphasize their individual perspective, understanding their self to be the only reason for their statements. Our finding that older women view themselves as lacking any of the biases others might perceive them as having or they see others as having are in line with the idea of the bias blind spot, whereby we see ourselves as being invulnerable to biases (McPherson Frantz, 2006). We find indications that there is a blind spot for the gender relatedness of own interest in technology, but an awareness of the age-relatedness of competence with technology.

### The Connection Between Age and Technology Shows a Correspondence Between the Perception of Oneself and of Others

We found that older women see their age as a reason for their (comparatively low) competence with technology. Thus, older women apparently do take biases into account when evaluating their competence with technology. They find new technologies to not be as intuitive to them as they might be because they see how easily younger people seem to use them. The older women search for an explanation for their challenges, which they find in their age. Other studies have also displayed older people’s hesitance to digital technology usage (Ball et al., 2017). Moreover, we put this in line with other studies demonstrating how older people use age as an explanatory factor for challenges they are faced with when using digital technologies (Stine-Morrow et al., 2006). This is why we assume that, contrary to the gender biases for interest in technology, there is not a discrepancy between the perception of themselves and others on competence with technology but rather a correspondence.

### Sense-Making as a Reason for Corresponding Evaluations of Competence With Technology in Oneself and in Others

Our findings are in line with the sense-making process, in which a person connects other people’s attitude and behavior ultimately with each other (Jones and Harris, 1967). With regard to themselves, people rely on their self-perception, therefore making conclusions about themselves based on their explicit behavior (Bem, 1972). From their self-perception, the women declare that their interest comes from their individual innate state; this self-perception arises from self-observation of one’s own behavior including interactions with the environment. Here, interest is a self-perception formed over time and is different from the perception of others. With regard to others, personality models (Hassabis et al., 2013) are constructed. Brain researchers have found that, when people make assumptions about other people’s behavior, a “mental simulation” (Hassabis et al., 2013, p. 1979) takes place in which the evaluating person makes a connection between personal experiences and future predictions:

“Both the construction and application of personality models are a key component of social processing, because these models are essential for predicting and comprehending the behavior of others. Identifying trait tendencies in others relies on an ability to accurately read and interpret social cues, then linking these to broader cognitive and behavioral tendencies” (Hassabis et al., 2013, p. 1979).

We found this interpersonal correspondence with the interviewed women, who ultimately assumed that women older than themselves must be less experienced in handling technology than they are because of the connection they make between technological competence and age. Other likely aspects – which could include interest in and talent for technology usage – were left out of the consideration. The interpretation of other people’s possible behavior is solely linked to one’s own reasoning for a certain behavior; in this case, people’s age is used as an explanation for their technological competence.

### Limitations

It should be emphasized at this juncture that the authors are aware that the social categories of gender and age are not the only realities for these women. Empirical research cannot be of infinite complexity, which is a known methodological problem, and the selection of categories is in itself a conscious evaluation process performed for research purposes (Schnicke, 2014). With respect to this study, it has been kept in mind that “any analysis of sexuality, power and gender must recognize the importance and interactive nature of their local, national and global contexts and the multiple and intersecting nature of the power relationships that can shape our identities, beliefs and behavior” (Jónasdóttir et al., 2011, p. 2). Hence, future research might include more or other social categories in combination with technology to better understand the impact of technology on social realities.

Moreover, to understand how the categories of gender and age in relation to technology work for combinations other than the ones we have used here, interviews with younger women, older men, or younger men could provide additional interesting insights. Investigating the perspectives of those groups would be an insightful contrast to our study – in relation to gender and/or in relation to age. However, such studies, including ours, are at risk of reproducing gender stereotypes and communicate stereotypes in a research context (Bruckmüller et al., 2012). We would like to highlight that the interpretation of such research needs to clearly point out the nature and influence of stereotypes versus actual behavior and competence.

In our study, as it is common in interview studies, the participants’ sentences, responses, and statements are influenced by the interview question being asked (Morrow, 2005). Moreover, the sample size is smaller than in most quantitative studies. Future research is needed to replicate and test the generalizability of our findings.

### Implications: Technologies Need to Be More Inclusive

For older women, the stereotypes of their gender and of their age are incongruent with stereotypes of both technological interest and technological competence. With our study, we provide a better understanding of older women’s perspectives on technology usage and competence.

With regards to gender and interest in technology, the older women repeat stereotypes and, hence, they reinforce them. By reproducing stereotypes – on themselves and on others – other people and future generations are influenced to replicate these stereotypes as well. The influence can unfold beyond the social category of older women: reinforcing the gender perspective could influence younger women to feel they are not expected to be interested in technology – or specific forms of technology – because it does not fit the gender stereotype; reinforcing the age perspective could influence older men to feel they are not supposed to put effort in learning new technologies because they would not be competent enough. With their stereotypical attributions, the women interfere with a more differentiated perception of interest in and competence with technology, which influences the relationship between discourse and behavior, especially for women and the older generation.

We intend to contribute to stereotype and self-perception literature by showing how a social group being negatively stereotyped taps into the repetition and eventually reproduction of the stereotypes discriminating them. Our findings on stereotyping in technology interaction can encourage future research on intersectionality. We intend to fuel the discussion to include technology as a social component influencing the intertwining of social categories. Our study suggests that technologies can be assessed as a depiction of existing gendered stereotypes. Therefore, we call for additional social science studies with technology-related research questions that can provide a better understanding of the role of technology for intersectionality. For example, the influence of Artificial Intelligence (AI) on a society as a whole and on human beings individually would be of particular interest. We hope our findings and thoughts on the intersecting influence of technology with social categories can inspire future research on the mutual influence of the various aspects of digitized systems and people.

Our research also has important implications for practice. Whether someone feels relegated or not, an inclusive society needs to involve all of us. The more digital technologies enable or even authorize people’s connection to society, the more crucial it will become to ensure accessibility for older women. This can be accomplished by sparking interest in new technologies and by supporting the competence level thereof. Older women can get support and acquire skills for using technology via digital literacy guidance. Role models in technology fields can have a positive impact, as shown by Herrmann et al. (2016), because people may identify with the presented rode model, and importantly, they identify best when the role model appears to be similar to themselves. Our findings go beyond previous ideas for actions: With the aim to increase interest of technology – such that our study implies that older women’s interest can be caught better when they are addressed individually than when they are addressed referring to their gender and age – as the older women reported see their individual preferences as explanations for their interest. With the aim to increase competence of technology, age-related role models could indeed be useful because the older women report age stereotypes as a justification for their perceived level of competence of technology such that a role model in their age may convince them that they are competent.

Our study aims at giving older women’s voices a wider audience and emphasizing that their voices have to be considered in technology development and policies. Given the ever-evolving process of automation and digitization, all groups should not only be guaranteed access to technology but should also influence technological development. Our research illuminated various examples of different ways of technology usage by older women. Thus, our study provides insights into older women’s individual ideas of incorporating technological products into their daily life. These numerous ways of usage can be considered a starting point for future developments of digitized products as well as studies on older women’s individual engagement. To ensure inclusive technology development, technological products need to be accessible and intuitive to older women in the first place. With smartphones functioning as an extension of our body and mind, their features need to consider the specifics of the end-user, and do so with regard to gender and age just as well. For the programmers, this includes considering distinct gesture and motor skills of older women, and taking into account their specific use of new technology as well as their various types of interests and competence levels.

## Conclusion

What kind of person do programmers have in mind when designing something that should be intuitively usable? And who decides how technology should be built so that it can be used intuitively? Here is where technology adds to the intersectionality of gender and age: digital technologies are powerful, and the people with influence on the designing end and competence on the user side are close to that power. This power sequence of technological influence affects the development of technology as older women often are neither impacting the direction of new technologies nor are they taken into account of shaping it. By considering gender and age in the development of digital technology, we can facilitate a more inclusive society.

## Data Availability Statement

The datasets generated for this study are available on request to the corresponding author.

## Ethics Statement

Ethical review and approval was not required for the study on human participants in accordance with the local legislation and institutional requirements. Written informed consent for participation was not required for this study in accordance with the national legislation and the institutional requirements. Oral informed consent for participation and approval by the participants was carried out before the interview and is recorded.

## Author Contributions

AG and SH contributed to the design and implementation of the research, to the analysis of the results and to the writing of the manuscript.

## Conflict of Interest

The authors declare that the research was conducted in the absence of any commercial or financial relationships that could be construed as a potential conflict of interest.

## Footnotes

1 This article refers to the study’s participant group as ‘older women’ which is only meant in a descriptive sense because of the relevant age category and is not meant to be discriminatory.

## APPENDIX

### Interview Guide

1. Introductory questions
  • How old are you and in what year were you born?
  • In what kind of relationship are you and since when?
  • What is your educational background?
  • What exactly was your job title?
  • Since when are you retired/in pension?
  • What does your everyday life look like today?
  • Do you have a volunteering position?
2. Familiarity with technology
  (a) General
    • How would you describe your knowledge about technology?
  (b) On the job
    • What technological equipment did you use in your job?
    • Who taught you how to use it?
  (c) In everyday life
    • What kind of technology do you use in everyday life?
    • Who taught you that?
    • Since when?
    • Where did you get the technology?
    • What kind of technology do you use for your hobbies/leisure/volunteering?
  (d) Personal opinion
    • How do you personally evaluate technologies, and specifically digital technologies?
3. Device of their choice
  (a) Presentation
  (b) Purpose
  (c) Demonstration
  (d) Clarifications
    • What position does this device have in your life?
    • What do you think about the device you chose?
    • Which applications/programs on the device do you use?
4. Situational questions
  • How do you think your environment perceives your technical behavior? And the technical behavior of your husband/partner?
  • What role does technology play in conversations within your circle of acquaintances?
  • What differences do you see in how men vs. women deal with technology?
  • What stereotypes about women and technology are you aware of?
  • How do you feel as a woman your age in relation to technology/society/your partner?
  • How does society perceive people your age? And women your age?
